# 4-Chloro­phenyl 4-methyl­benzoate

**DOI:** 10.1107/S1600536808021697

**Published:** 2008-07-16

**Authors:** B. Thimme Gowda, Sabine Foro, K. S. Babitha, Hartmut Fuess

**Affiliations:** a Department of Chemistry, Mangalore University, Mangalagangotri 574 199, Mangalore, India; b Institute of Materials Science, Darmstadt University of Technology, Petersenstrasse 23, D-64287 Darmstadt, Germany

## Abstract

The crystal structure of the title compound (4CP4MBA), C_14_H_11_ClO_2_, resembles those of 3-chloro­phenyl 4-methyl­benzoate (3CP4MBA), 4-methyl­phenyl 4-methyl­benzoate (4MP4MBA), 4-methyl­phenyl 4-chloro­benzoate (4MP4CBA) and other aryl benzoates with similar bond parameters. The dihedral angle between the benzene rings in 4CP4MBA is 63.89 (8)°, compared with 71.75 (7)° in 3CP4MBA, 63.57 (5)° in 4MP4MBA and 51.86 (4)° in 4MP4CBA. In the crystal structure of the title compound, the mol­ecules are linked into an infinite chain along the *a* axis *via* C—H—O hydrogen bonds.

## Related literature

For related literature, see: Gowda *et al.* (2007 ▶); Gowda, Foro, *et al.* (2008 ▶); Gowda, Svoboda *et al.* (2008 ▶); Nayak & Gowda (2008 ▶).
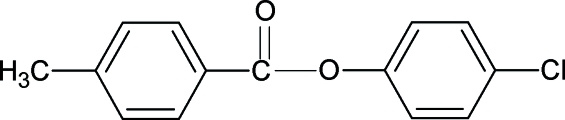


## Experimental

### 

#### Crystal data

C_14_H_11_ClO_2_
*M* *_r_* = 246.68
Monoclinic, 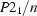

*a* = 6.048 (2) Å
*b* = 7.559 (2) Å
*c* = 26.487 (5) Å
β = 95.68 (4)°
*V* = 1205.0 (6) Å^3^
*Z* = 4
Cu *K*α radiation
μ = 2.69 mm^−1^
*T* = 299 (2) K
0.65 × 0.60 × 0.45 mm

#### Data collection

Enraf–Nonius CAD-4 diffractometer
Absorption correction: ψ scan (North *et al.*, 1968 ▶) *T*_min_ = 0.216, *T*_max_ = 0.298
2872 measured reflections
2141 independent reflections
1968 reflections with *I* > 2σ(*I*)
*R*_int_ = 0.053
3 standard reflections frequency: 120 min intensity decay: 1.5%

#### Refinement

*R*[*F* ^2^ > 2σ(*F* ^2^)] = 0.092
*wR*(*F* ^2^) = 0.313
*S* = 1.51
2141 reflections
156 parameters
H-atom parameters constrained
Δρ_max_ = 0.53 e Å^−3^
Δρ_min_ = −0.94 e Å^−3^

### 

Data collection: *CAD-4-PC* (Enraf–Nonius, 1996 ▶); cell refinement: *CAD-4-PC*; data reduction: *REDU4* (Stoe & Cie, 1987 ▶); program(s) used to solve structure: *SHELXS97* (Sheldrick, 2008 ▶); program(s) used to refine structure: *SHELXL97* (Sheldrick, 2008 ▶); molecular graphics: *PLATON* (Spek, 2003 ▶); software used to prepare material for publication: *SHELXL97*.

## Supplementary Material

Crystal structure: contains datablocks I, global. DOI: 10.1107/S1600536808021697/is2314sup1.cif

Structure factors: contains datablocks I. DOI: 10.1107/S1600536808021697/is2314Isup2.hkl

Additional supplementary materials:  crystallographic information; 3D view; checkCIF report

## Figures and Tables

**Table 1:** Hydrogen-bond geometry (Å, °)

*D*—H⋯*A*	*D*—H	H⋯*A*	*D*⋯*A*	*D*—H⋯*A*
C2—H2⋯O2^i^	0.93	2.51	3.212 (3)	132

Symmetry code: (i) 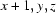.

**Fig. 1.**
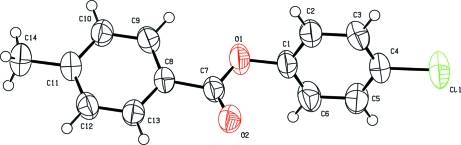
Molecular structure of the title compound, showing the atom labeling. Displacement ellipsoids are drawn at the 50% probability level. H atoms are represented as small spheres of arbitrary radius.

**Fig. 2.**
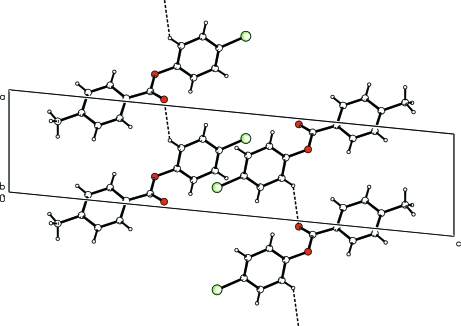
Molecular packing of the title compound, viewed along the b axis.

## supplementary crystallographic information

### Comment

In the present work, as part of a study of the substituent effects on the solid state structures of aryl benzoates (Gowda *et al.* 2007; Gowda, Foro, *et al.*, 2008; Gowda, Svoboda *et al.*, 2008), the structure of 4-chlorophenyl 4-methylbenzoate (4CP4MBA) has been determined. The structure of 4CP4MBA (Fig. 1) is similar to those of 3-chlorophenyl 4-methylbenzoate (3CP4MBA)(Gowda, Foro *et al.*, 2008), 4-methylphenyl 4-methylbenzoate (4MP4MBA)(Gowda *et al.*, 2007), 4-methylphenyl 4-chlorobenzoate (4MP4CBA) (Gowda, Svoboda *et al.*, 2008) The bond parameters in 4CP4MBA are similar to those in 3CP4MBA, 4MP4MBA, 4MP4CBA and other aryl benzoates. The dihedral angle between the benzene and benzoyl rings in 4CP4MBA is 63.89 (8)°, compared to the values of 71.75 (7)° in 3CP4MeBA, 63.57 (5)° in 4MP4MBA and 51.86 (4)° in 4MP4CBA. The molecules in the crystal structure of 4CP4MBA are packed into chains *via* C—H—O hydrogen bonds (Table 1 and Fig. 2).

### Experimental

The title compound was prepared according to a literature method (Nayak & Gowda, 2008). The purity of the compound was checked by determining its melting point. It was characterized by recording its infrared and NMR spectra (Nayak & Gowda, 2008). Single crystals of the title compound used in X-ray diffraction studies were obtained by slow evaporation of its ethanol solution.

### Refinement

All H atoms were included in the riding-model approximation, with C—H = 0.93–0.96 Å, and with *U*_iso_(H) = 1.2*U*_eq_(C).

### Crystal data

**Table:**

C_14_H_11_ClO_2_	*F*_000_ = 512
*M**_r_* = 246.68	*D*_x_ = 1.360 Mg m^−^^3^
Monoclinic, *P*2_1_/*n*	Cu *K*α radiation λ = 1.54180 Å
Hall symbol: -P 2yn	Cell parameters from 25 reflections
*a* = 6.048 (2) Å	θ = 3.4–16.9º
*b* = 7.559 (2) Å	µ = 2.69 mm^−^^1^
*c* = 26.487 (5) Å	*T* = 299 (2) K
β = 95.68 (4)º	Prism, colourless
*V* = 1205.0 (6) Å^3^	0.65 × 0.60 × 0.45 mm
*Z* = 4	

### Data collection

**Table:**

Enraf–Nonius CAD-4 diffractometer	*R*_int_ = 0.053
Radiation source: fine-focus sealed tube	θ_max_ = 67.0º
Monochromator: graphite	θ_min_ = 3.4º
*T* = 299(2) K	*h* = −7→2
ω/2θ scans	*k* = −9→0
Absorption correction: ψ scan(North *et al.*, 1968)	*l* = −31→31
*T*_min_ = 0.216, *T*_max_ = 0.298	3 standard reflections
2872 measured reflections	every 120 min
2141 independent reflections	intensity decay: 1.5%
1968 reflections with *I* > 2σ(*I*)	

### Refinement

**Table:**

Refinement on *F*^2^	Hydrogen site location: inferred from neighbouring sites
Least-squares matrix: full	H-atom parameters constrained
*R*[*F*^2^ > 2σ(*F*^2^)] = 0.092	*w* = 1/[σ^2^(*F*_o_^2^) + (0.2*P*)^2^] where *P* = (*F*_o_^2^ + 2*F*_c_^2^)/3
*wR*(*F*^2^) = 0.313	(Δ/σ)_max_ = 0.001
*S* = 1.51	Δρ_max_ = 0.53 e Å^−^^3^
2141 reflections	Δρ_min_ = −0.94 e Å^−^^3^
156 parameters	Extinction correction: SHELXL97 (Sheldrick, 2008), Fc^*^=kFc[1+0.001xFc^2^λ^3^/sin(2θ)]^-1/4^
Primary atom site location: structure-invariant direct methods	Extinction coefficient: 0.048 (9)
Secondary atom site location: difference Fourier map	

### Special details

**Table:**

Geometry. All e.s.d.'s (except the e.s.d. in the dihedral angle between two l.s. planes) are estimated using the full covariance matrix. The cell e.s.d.'s are taken into account individually in the estimation of e.s.d.'s in distances, angles and torsion angles; correlations between e.s.d.'s in cell parameters are only used when they are defined by crystal symmetry. An approximate (isotropic) treatment of cell e.s.d.'s is used for estimating e.s.d.'s involving l.s. planes.
Refinement. Refinement of *F*^2^ against ALL reflections. The weighted *R*-factor *wR* and goodness of fit *S* are based on *F*^2^, conventional *R*-factors *R* are based on *F*, with *F* set to zero for negative *F*^2^. The threshold expression of *F*^2^ > σ(*F*^2^) is used only for calculating *R*-factors(gt) *etc*. and is not relevant to the choice of reflections for refinement. *R*-factors based on *F*^2^ are statistically about twice as large as those based on *F*, and *R*- factors based on ALL data will be even larger.

### Fractional atomic coordinates and isotropic or equivalent isotropic displacement parameters (Å^2^)

**Table:**

	*x*	*y*	*z*	*U*_iso_*/*U*_eq_	
Cl1	0.75384 (13)	0.28660 (13)	0.53235 (3)	0.0895 (6)	
O1	0.2922 (3)	0.3273 (3)	0.32780 (7)	0.0708 (7)	
O2	0.0565 (3)	0.5372 (3)	0.34855 (6)	0.0655 (7)	
C1	0.3932 (4)	0.3235 (3)	0.37781 (9)	0.0577 (7)	
C2	0.5981 (4)	0.4028 (4)	0.38707 (10)	0.0627 (8)	
H2	0.6612	0.4629	0.3614	0.075*	
C3	0.7081 (4)	0.3910 (3)	0.43527 (11)	0.0643 (8)	
H3	0.8467	0.4436	0.4425	0.077*	
C4	0.6124 (5)	0.3022 (3)	0.47205 (10)	0.0618 (8)	
C5	0.4096 (5)	0.2231 (4)	0.46293 (11)	0.0697 (8)	
H5	0.3473	0.1627	0.4886	0.084*	
C6	0.2981 (4)	0.2344 (4)	0.41478 (11)	0.0693 (8)	
H6	0.1596	0.1815	0.4078	0.083*	
C7	0.1213 (3)	0.4406 (3)	0.31755 (8)	0.0522 (7)	
C8	0.0302 (3)	0.4314 (3)	0.26348 (8)	0.0492 (7)	
C9	0.1415 (4)	0.3453 (3)	0.22735 (9)	0.0571 (7)	
H9	0.2781	0.2919	0.2366	0.068*	
C10	0.0495 (4)	0.3389 (3)	0.17768 (10)	0.0591 (7)	
H10	0.1266	0.2834	0.1535	0.071*	
C11	−0.1566 (4)	0.4140 (3)	0.16318 (9)	0.0549 (7)	
C12	−0.2636 (4)	0.5008 (3)	0.19962 (10)	0.0605 (7)	
H12	−0.4012	0.5527	0.1905	0.073*	
C13	−0.1715 (4)	0.5122 (3)	0.24905 (9)	0.0579 (7)	
H13	−0.2446	0.5741	0.2728	0.069*	
C14	−0.2612 (5)	0.4005 (4)	0.10953 (11)	0.0718 (8)	
H14A	−0.1757	0.3214	0.0908	0.086*	
H14B	−0.2651	0.5154	0.0940	0.086*	
H14C	−0.4099	0.3559	0.1094	0.086*	

### Atomic displacement parameters (Å^2^)

**Table:**

	*U*^11^	*U*^22^	*U*^33^	*U*^12^	*U*^13^	*U*^23^
Cl1	0.0931 (9)	0.1079 (9)	0.0595 (8)	−0.0047 (4)	−0.0318 (5)	0.0080 (3)
O1	0.0735 (12)	0.0844 (13)	0.0496 (12)	0.0203 (9)	−0.0187 (8)	−0.0115 (9)
O2	0.0671 (12)	0.0793 (12)	0.0480 (11)	0.0089 (8)	−0.0050 (7)	−0.0095 (8)
C1	0.0570 (13)	0.0664 (13)	0.0460 (14)	0.0066 (9)	−0.0130 (9)	−0.0050 (10)
C2	0.0586 (14)	0.0753 (15)	0.0522 (15)	−0.0014 (10)	−0.0054 (10)	0.0072 (12)
C3	0.0543 (13)	0.0703 (15)	0.0644 (16)	−0.0030 (10)	−0.0135 (10)	0.0029 (12)
C4	0.0653 (15)	0.0640 (14)	0.0518 (15)	0.0026 (9)	−0.0152 (10)	0.0020 (10)
C5	0.0730 (16)	0.0801 (17)	0.0532 (16)	−0.0130 (12)	−0.0069 (12)	0.0089 (12)
C6	0.0609 (15)	0.0791 (16)	0.0637 (17)	−0.0128 (11)	−0.0148 (11)	−0.0027 (13)
C7	0.0480 (12)	0.0595 (13)	0.0471 (13)	−0.0029 (8)	−0.0061 (9)	−0.0003 (9)
C8	0.0506 (11)	0.0511 (11)	0.0439 (13)	−0.0028 (7)	−0.0058 (8)	0.0004 (8)
C9	0.0525 (13)	0.0604 (12)	0.0557 (14)	0.0063 (9)	−0.0076 (9)	−0.0049 (10)
C10	0.0635 (15)	0.0665 (14)	0.0458 (13)	0.0082 (10)	−0.0025 (10)	−0.0061 (10)
C11	0.0635 (13)	0.0499 (11)	0.0481 (14)	−0.0014 (8)	−0.0103 (10)	0.0022 (9)
C12	0.0555 (13)	0.0675 (14)	0.0556 (14)	0.0098 (9)	−0.0085 (10)	0.0043 (11)
C13	0.0607 (14)	0.0665 (14)	0.0452 (13)	0.0081 (9)	−0.0012 (9)	−0.0032 (10)
C14	0.0862 (19)	0.0737 (16)	0.0512 (16)	0.0040 (12)	−0.0150 (12)	−0.0004 (12)

### Geometric parameters (Å, °)

**Table:**

Cl1—C4	1.740 (3)	C8—C13	1.384 (3)
O1—C7	1.350 (3)	C8—C9	1.386 (3)
O1—C1	1.403 (3)	C9—C10	1.378 (3)
O2—C7	1.193 (3)	C9—H9	0.9300
C1—C6	1.362 (4)	C10—C11	1.389 (3)
C1—C2	1.376 (4)	C10—H10	0.9300
C2—C3	1.383 (4)	C11—C12	1.380 (4)
C2—H2	0.9300	C11—C14	1.501 (3)
C3—C4	1.359 (4)	C12—C13	1.374 (4)
C3—H3	0.9300	C12—H12	0.9300
C4—C5	1.364 (4)	C13—H13	0.9300
C5—C6	1.385 (4)	C14—H14A	0.9600
C5—H5	0.9300	C14—H14B	0.9600
C6—H6	0.9300	C14—H14C	0.9600
C7—C8	1.484 (3)		
			
C7—O1—C1	117.12 (18)	C13—C8—C7	118.57 (19)
C6—C1—C2	121.6 (3)	C9—C8—C7	122.0 (2)
C6—C1—O1	120.9 (2)	C10—C9—C8	119.8 (2)
C2—C1—O1	117.4 (2)	C10—C9—H9	120.1
C1—C2—C3	118.6 (2)	C8—C9—H9	120.1
C1—C2—H2	120.7	C9—C10—C11	121.1 (2)
C3—C2—H2	120.7	C9—C10—H10	119.4
C4—C3—C2	119.6 (2)	C11—C10—H10	119.4
C4—C3—H3	120.2	C12—C11—C10	118.1 (2)
C2—C3—H3	120.2	C12—C11—C14	120.8 (2)
C3—C4—C5	121.9 (3)	C10—C11—C14	121.1 (2)
C3—C4—Cl1	119.1 (2)	C13—C12—C11	121.5 (2)
C5—C4—Cl1	119.0 (2)	C13—C12—H12	119.2
C4—C5—C6	118.9 (3)	C11—C12—H12	119.2
C4—C5—H5	120.5	C12—C13—C8	119.9 (2)
C6—C5—H5	120.5	C12—C13—H13	120.0
C1—C6—C5	119.4 (2)	C8—C13—H13	120.0
C1—C6—H6	120.3	C11—C14—H14A	109.5
C5—C6—H6	120.3	C11—C14—H14B	109.5
O2—C7—O1	123.2 (2)	H14A—C14—H14B	109.5
O2—C7—C8	125.2 (2)	C11—C14—H14C	109.5
O1—C7—C8	111.57 (19)	H14A—C14—H14C	109.5
C13—C8—C9	119.5 (2)	H14B—C14—H14C	109.5
			
C7—O1—C1—C6	79.0 (3)	O2—C7—C8—C13	−13.8 (3)
C7—O1—C1—C2	−105.3 (3)	O1—C7—C8—C13	167.3 (2)
C6—C1—C2—C3	−0.2 (4)	O2—C7—C8—C9	165.9 (2)
O1—C1—C2—C3	−175.8 (2)	O1—C7—C8—C9	−12.9 (3)
C1—C2—C3—C4	0.1 (4)	C13—C8—C9—C10	−0.8 (3)
C2—C3—C4—C5	0.1 (4)	C7—C8—C9—C10	179.5 (2)
C2—C3—C4—Cl1	179.36 (19)	C8—C9—C10—C11	−1.5 (4)
C3—C4—C5—C6	−0.2 (5)	C9—C10—C11—C12	2.2 (4)
Cl1—C4—C5—C6	−179.4 (2)	C9—C10—C11—C14	−177.4 (2)
C2—C1—C6—C5	0.1 (4)	C10—C11—C12—C13	−0.5 (4)
O1—C1—C6—C5	175.6 (2)	C14—C11—C12—C13	179.1 (2)
C4—C5—C6—C1	0.1 (5)	C11—C12—C13—C8	−1.8 (4)
C1—O1—C7—O2	1.1 (3)	C9—C8—C13—C12	2.5 (4)
C1—O1—C7—C8	179.94 (19)	C7—C8—C13—C12	−177.8 (2)

### Hydrogen-bond geometry (Å, °)

**Table:**

*D*—H···*A*	*D*—H	H···*A*	*D*···*A*	*D*—H···*A*
C2—H2···O2^i^	0.93	2.51	3.212 (3)	132

Symmetry codes: (i) *x*+1, *y*, *z*.
